# The association between lifetime smoking exposure and breast cancer mortality – results from a Norwegian cohort

**DOI:** 10.1002/cam4.304

**Published:** 2014-07-30

**Authors:** Eivind Bjerkaas, Ranjan Parajuli, Anders Engeland, Gertraud Maskarinec, Elisabete Weiderpass, Inger Torhild Gram

**Affiliations:** 1 Department of Community Medicine, Faculty of Health Sciences, UiT The Arctic University of NorwayN-9037, Tromsø, Norway; 2 Division of Epidemiology, Department of Pharmacoepidemiology, Norwegian Institute of Public HealthOslo, Norway; 3 Department of Global Public Health and Primary Care, University of BergenBergen, Norway; 4 University of Hawaii Cancer CenterHonolulu, Hawaii; 5 Department of Medical Epidemiology and Biostatistics, Karolinska InstitutetStockholm, Sweden; 6 Department of Genetic Epidemiology, Folkhälsan Research Center, Samfundet FolkhälsanHelsinki, Finland; 7 Department of Research, Cancer Registry of NorwayOslo, Norway; 8 Norwegian Centre for Integrated Care and Telemedicine, University Hospital of North NorwayTromsø, Norway

**Keywords:** Breast cancer, cohort study, CONOR, mortality, smoking

## Abstract

Several recent cohort studies have found an association between smoking and breast cancer, but the association between lifetime smoking exposure and breast cancer mortality is less well described. We examined whether smoking before breast cancer diagnosis is a predictor of breast cancer mortality in a large cohort with more than 4.1 million years of follow-up, with a special focus on women who initiated smoking before first childbirth. Information on smoking status was collected before breast cancer diagnosis and used to estimate hazard ratios (HRs) and corresponding 95% confidence intervals (CIs) of breast cancer mortality in a cohort of 302,865 Norwegian women with 1106 breast cancer deaths. Women were enrolled between 1974 and 2003 and followed up through linkages to national registries until 31 December 2007. We found that breast cancer mortality was slightly but significantly increased for current (HR = 1.15, 95% CI 1.01–1.32) and ever (HR = 1.15, 95% CI 1.02–1.30) smokers as compared to never smokers. No statistically significantly increased mortality was found for women who initiated smoking before first childbirth, and no dose-response association was revealed for any of the different measures of smoking exposure. A large proportion of heavy smokers may have died from other causes than breast cancer during follow-up, possibly diluting our results. This study found that lifetime smoking exposure had a significantly increased risk of breast cancer mortality compared with never smokers.

## Introduction

In the past 30 years in Norway, breast cancer incidence has almost doubled while mortality has been stable, or decreased, in the same period 1–4. In 2011, 3094 women were diagnosed with breast cancer in Norway, and 605 women died from the disease the same year 3. This difference in incidence and mortality reflects the good prognosis of breast cancer, especially when diagnosed at an early stage. Nevertheless, breast cancer remains the leading cause of cancer death among women worldwide 5.

The International Agency for Research on Cancer recently classified cigarette smoking as possibly carcinogenic to the human breast 6, but the relationship between smoking and breast cancer is still controversial 7–10. Several large prospective cohort studies have found that smoking may cause breast cancer particularly in women who smoke for a long time, in those who smoke many cigarettes per day, and in those who initiated smoking before the first childbirth 11–20. The association between lifetime smoking exposure and breast cancer mortality is uncertain and not well described in the literature 21–24. Most previous studies have assessed smoking status at breast cancer diagnosis, finding an increased risk among current but not among former smokers 25–32, possibly underestimating the effect of lifetime smoking exposure among former smokers 23,24.

The proportion of women who smoke daily in Norway increased from 23% in 1954 to 37% in 1970, before stabilizing at around 32% for the rest of the century 33. This high percentage, together with an increased proportion of women smoking before the first childbirth in younger birth cohorts 19, represents a suitable setting for studying the association between lifetime smoking exposure and breast cancer mortality in this large pooled cohort.

Cancer mortality is a function of incidence influenced by exposure to lifetime risk factors, inherited factors and survival after diagnosis 34. Exposure to risk factors in the period before breast cancer diagnosis is likely to influence the development of the disease 35, and modifiable risk factors from this period are important to identify. In this study, we used information collected before breast cancer diagnosis to examine the potential importance of smoking before diagnosis as a predictor of breast cancer mortality. Smoking in the time period from menarche to first childbirth has emerged as a risk factor for breast cancer in many epidemiological studies 7,8,14,16–19,36, and animal studies have confirmed that mammary cells are more vulnerable to chemical carcinogens in this time period 37. Our particular interest was whether the increased breast cancer risk for female smokers who initiate smoking before first childbirth increases the risk of dying from breast cancer later in life 19.

## Materials and Methods

### Study population

The study population comprised Norwegian women recruited into three prospective cohort studies conducted by the National Health Screening Service (now the Norwegian Institute of Public Health): the Norwegian Counties Study (1974–1988), the 40 Years Study (1985–1999) and the Cohort of Norway (CONOR) Study (1994–2003). Selection of participants in these studies was usually based on year of birth and residence (municipality or county). A total of 330,342 women were eligible before exclusion, and if any women participated in more than one study only the first record was used. All women with a cancer diagnosis prior to start of follow-up (*n* = 7138), women without information on smoking status (*n* = 2808), education level (*n* = 6913), body mass index (BMI, *n* = 2478), and physical activity (*n* = 4207) were excluded from the present analysis, leaving 302,865 women in the analytical cohort.

The response rate in the three studies varied from 56% to 88% 38. All participants recruited as from 1994 gave written informed consent to participate in the studies; before 1994 returning the completed questionnaire was considered sufficient as acceptance to participate in the studies. This study was approved by the Regional Committee for Medical Research Ethics South-East, Norway.

The design and protocol of the three studies were similar, although some modifications regarding smoking, level of physical activity and other lifestyle factors were made in the questionnaires at different time periods 39–41. All studies had a baseline questionnaire which included detailed assessments of smoking habits, level of physical activity, and other lifestyle factors, but not hormone replacement use. In addition, questions on alcohol consumption were only included from 1994 onwards. The wording of the questionnaires was standardized when the CONOR collaboration was initiated in 1994 41.

### Exposure information

After receiving specified variables from the primary data of each study, we created a standardized database for the pooled analysis based on the CONOR database. The smoking questions were similar, but not identical, across all surveys. Based on their responses at study enrolment, participants were classified as never, former, or current smokers, which remained the classification throughout the follow-up period. Current and former smokers were considered ever smokers, and were further classified by age at smoking initiation, smoking duration in years, average number of cigarettes smoked per day and number of pack-years (i.e., number of cigarettes smoked per day, divided by 20, multiplied by the smoking duration in years). Only the CONOR Study had a specific question related to age at smoking initiation. In the other two studies we calculated this variable for both current smokers (age at enrolment minus smoking duration in years) and former smokers (age at quitting smoking minus smoking duration in years). For parous women, the variable “smoking duration before first childbirth” was calculated as age at first childbirth minus age at smoking initiation. All participants who were neither current nor former smokers were classified as never smokers, and never smokers constitute the reference group throughout this paper, unless otherwise noted.

Participants were categorized into three groups based on their level of physical activity at enrolment: sedentary (reading, watching television, sedentary activity, walking or bicycling, <4 h per week), moderate (walking, bicycling, and/or similar activities ≥4 h per week), and heavy (light sports or heavy gardening ≥4 h per week, heavy exercise or daily competitive sports). The most recent information regarding duration of education obtained from Statistics Norway was used to assign participants to one of three categories according to duration of education: <10, 10–12, ≥13 years. Information on number of children and age at first childbirth was also obtained through linkages to Statistics Norway. As questions on alcohol consumption were only included from 1994 onwards, information on alcohol consumption was missing in 62% of the women in the analytical cohort.

### Follow-up and endpoints

We followed all participants through linkages with the Cancer Registry of Norway, the Norwegian Cause of Death Registry, and the Central Population Register using the unique 11-digit personal identification number to identify all cancer cases, deaths and emigrations. Information about tumor stage at diagnosis, or treatment, was not considered in our analysis. These national registries are accurate and may be regarded as virtually complete 42. The start of follow-up was set to January 1 the year following completion of the baseline questionnaire. The International Classification of Diseases (ICD-9/ICD-10) codes were used to identify breast cancer as the underlying cause of death in the registries. To correct for errors and mistaken conclusions drawn by the physician, rules from the World Health Organization are used to ensure correct classification on the basis of the death certificate 43,44.

### Statistical analysis

We used Cox proportional hazard models (with age as the underlying time scale) to estimate the multivariate-adjusted hazard ratios (HRs) with 95% confidence intervals (CIs) of breast cancer mortality associated with different measures of smoking exposure (age at smoking initiation [≤19, 20–24 and ≥25 years], smoking duration [≤10, 11–20, and ≥21 years], number of cigarettes smoked per day [≤5, 6–10, ≥11 cigarettes], number of pack-years [≤5, 6–10, ≥11 pack-years], and for parous women smoking duration in relation to first childbirth [more than 1 year after first childbirth, around [i.e., 1 year before to 1 year after], >1–6 years before, ≥7 years before first childbirth]). Entry time in the statistical model was defined as age at enrolment, and exit time as age at death, emigration or the end of follow-up (31 December 2007), whichever occurred first.

The covariates included in the final models, decided a priori, were age at enrolment (continuous variable), duration of education (<10, 10–12, ≥13 years), number of children (0, 1–2, 3–4, ≥5), age at first childbirth (<20, 20–24, 25–29, ≥30 years), BMI (<25, 25–29, ≥30 kg/m^2^) and level of physical activity (sedentary, moderate, heavy). We analyzed the age- and multivariate-adjusted HRs with 95% CIs for breast cancer mortality according to the selected covariates included in the multivariate analysis.

The multivariate analysis in Tables 2 and 3 were stratified by the three studies. Further, we estimated the possible impact of differences in birth cohorts, and stratified the full cohort by birth year (≤1950>) for the multivariate analysis in Tables 2 and 3 (data not shown). Alcohol consumption was categorized as less than weekly (including teetotallers), weekly and more than weekly, and was included in the multivariate model in a subanalysis as an adjusting variable. We did tests for linear trends across the different levels of smoking exposures, including the reference category (Table 2), excluding never smokers (Table 3). The results were considered significant if the *P* value was <0.05, or if the CIs were outside 1.00. All *P* values are two sided. The analyses were done in STATA version 12.0 (StataCorp, College Station, TX), and SAS version 9.4 (SAS Institute Inc., Cary, NC).

## Results

At study enrolment, the mean age was 44 years and 59% of the 302,865 Norwegian women were ever smokers. During 14 years of median follow-up, we confirmed 1106 breast cancer deaths and 14,446 deaths by all other causes. Mean age at breast cancer death was 61 years in the Counties Study, 54 years in the 40 Years Study and 66 years in the CONOR Study (Table 1).

**Table 1:** Selected characteristics of the analytical cohort, stratified by studies, among 302,865 Norwegian women (1974–2003)

Characteristics	Counties Study	40 Years Study	CONOR Study	All
Study period	1974–1987	1985–1999	1994–2003	1974–2003
Person years of follow-up	1,075,997	2,577,627	510,690	4,164,314
Participants	41,573	199,729	61,563	302,865
Age^1^, mean, SD	40 ± 7	43 ± 5	48 ± 15	44 ± 9
Year of birth, median, (range)	1939 (1932–1944)	1951 (1948–1954)	1955 (1941–1960)	1951 (1946–1955)
Age at breast cancer diagnosis, mean, SD	58 ± 9	52 ± 7	59 ± 13	54 ± 9
Year of breast cancer diagnosis, median, (range)	1998 (1991–2003)	2002 (1998–2005)	2004 (2001–2005)	2002 (1997–2005)
Age at breast cancer death, mean, SD	61 ± 9	54 ± 8	66 ± 16	58 ± 10
Number of breast cancer deaths	405	624	77	1106
Ever daily smokers^1^ among breast cancer deaths (%)	54	62	53	58
Age at death, all causes^2^, mean, SD	65 ± 9	60 ± 13	78 ± 13	66 ± 14
Number of deaths, all causes^2^	5401	6471	3680	15,552
Ever daily smokers^2^ among dead from all causes^3^ (%)	66	67	46	61
Follow-up years, median	30	13	9	14
≥13 years of education, (%)	12	22	21	21
Number of children, mean, SD	2 ± 2	2 ± 1	2 ± 1	2 ± 1
Body mass index[Table-fn tf1-2], mean, (kg/m²)	24	24	25	25
Level of physical activity, heavy^1^,^3^ (%)	11	21	28	21
Smoking status^1^				
Never daily smokers (%)	46	39	44	41
Ever daily smokers^4^ (%)	54	61	56	59

A former smoker has been a daily smoker previously. *SD* standard deviation, *Range* interquartile range.

1 At enrollment.

2 Deaths by all causes includes deaths by breast cancer.

3 Heavy physical activity is defined as light sports or heavy gardening ≥4 h per week, heavy exercise or daily competitive sports.

4 Ever smoker: current and former smoker.

Table 2 shows that for women overall, significant dose-response associations with breast cancer mortality were observed for parity (inversely related: *P*_trend_ < 0.01), and were positively related with age at first childbirth (*P*_trend_ < 0.01) and with BMI (*P*_trend_ = 0.02). Information on alcohol consumption was only available for 36.9% of the women, and showed a nonsignificant 22% increased risk of breast cancer mortality for those drinking > weekly (HR = 1.22, 95% CI 0.73–2.03).

**Table 2:** Mulitvariate adjusted hazard ratios (HRs) and 95% confidence intervals (CIs) for breast cancer mortality^1^, stratified by studies, according to selected covariates

	Counties Study	BC cases	40 Years Study	BC cases	CONOR Study	BC cases	All studies	BC cases
Duration of education (years)								
<10	Ref.	176	Ref.	168	Ref.	26	Ref.	370
10–12	1.12 (0.90–1.38)	178	0.91 (0.75–1.11)	330	1.18 (0.69–2.28)	37	1.00 (0.86–1.14)	545
≥13	1.40 (1.02–1.93)	51	0.96 (0.75–1.23)	126	1.32 (0.64–2.71)	14	1.09 (0.91–1.31)	191
*P*_trend_	0.05		0.71		0.43		0.44	
Number of children								
0	Ref.	71	Ref.	79	Ref.	10	Ref.	160
1–2	0.55 (0.33–0.91)	177	0.50 (0.34–0.74)	356	0.44 (0.13–1.55)	40	0.50 (0.37–0.67)	573
3–4	0.41 (0.24–0.68)	135	0.40 (0.26–0.60)	179	0.37 (0.10–1.34)	25	0.38 (0.28–0.52)	339
≥5	0.26 (0.14–0.50)	22	0.29 (0.14–0.59)	10	0.19 (0.04–1.17)	2	0.25 (0.16–0.39)	34
*P*_trend_	<0.01		<0.01		0.09		<0.01	
Age at first childbirth^2^ (year)								
<20	Ref.	24	Ref.	61	Ref.	6	Ref.	91
20–24	1.29 (0.84–1.99)	153	1.15 (0.86–1.53)	238	0.84 (0.34–2.12)	21	1.17 (0.93–1.47)	412
25–29	1.40 (0.88–2.22)	103	1.44 (1.05–1.96)	160	0.94 (0.35–2.51)	17	1.40 (1.09–1.79)	280
≥30	1.61 (0.97–2.70)	125	1.84 (1.29–2.63)	165	2.09 (0.75–5.81)	33	1.82 (1.40–2.40)	323
*P*_trend_	0.07		<0.01		0.04		<0.01	
Body mass index^3^ (kg/m²)								
<25	Ref.	246	Ref.	389	Ref.	24	Ref.	659
25–29	1.07 (0.86–1.35)	146	1.11 (0.93–1.33)	245	1.73 (1.01–2.96)	38	1.12 (0.98–1.29)	429
≥30	1.32 (0.96–1.81)	63	1.07 (0.81–1.42)	76	2.17 (1.17–4.02)	25	1.22 (1.01–1.49)	164
*P*_trend_	0.02		0.33		0.01		0.02	
Physical activity^3^								
Sedentary	Ref.	87	Ref.	130	Ref.	40	Ref.	257
Moderate	1.01 (0.79–1.28)	277	0.93 (0.76–1.13)	416	0.80 (0.48–1.33)	25	1.00 (0.86–1.15)	718
Heavy	0.95 (0.66–1.39)	41	0.69 (0.52–0.92)	78	0.74 (0.38–1.47)	12	0.78 (0.63–0.97)	131
*P*_trend_	0.87		0.02		0.32		0.06	
Alcohol consumption^3^,^4^								
<Weekly^5^		NA	Ref.	42	Ref.	47	Ref.	89
Weekly		NA	1.78 (0.98–3.22)	15	0.42 (0.14–1.18)	4	1.05 (0.63–1.74)	19
>Weekly		NA	1.34 (0.72–2.50)	14	1.06 (0.41–2.77)	5	1.22 (0.73–2.03)	19
*P*_trend_			0.18		0.49		0.46	

Adjusted for age, education level, number of children, age at first childbirth, BMI, age at enrollment and physical activity.

Trend tests between the three or four levels of categories, including the reference category.

1 Deaths by breast cancer (*n* = 302,865 with 1106 cases).

2 Nulliparous (*n* = 36,523) not included.

3 At enrollment.

4 Only women with alcohol information included (*n* = 114,804).

5 Including teetotalers.

Table 3 shows that the overall results for former (HR = 1.14, CI 0.97–1.34) and current (HR = 1.15, 95% CI 1.01–1.32) smokers had similar increased risks for breast cancer mortality in the multivariate analysis compared with never smokers. For ever smokers a significantly increased risk was found among women who initiated smoking at ≥25 years of age (HR = 1.31, 95% CI 1.08–1.59), among those smoking 11–20 years of duration (HR = 1.20, 95% CI 1.03–1.40), and among those smoking ≥11 cigarettes per day (HR = 1.25, 95% CI 1.06–1.46). Parous women who initiated smoking 7 years or more before first childbirth had a 24% (HR = 1.24, 95% CI 0.98–1.58) nonsignificantly increased breast cancer mortality compared to never smokers. The overall results revealed no dose-response associations for any of the different measures of smoking exposure (age at smoking initiation, smoking duration, number of cigarettes smoked per day, number of pack-years, and smoking duration before first childbirth, all *P* for trends ≥0.05) and breast cancer mortality. One significant dose-response association with breast cancer mortality was observed in the Counties Study for number of cigarettes smoked per day (*P*_trend_ < 0.01), but no other consistent pattern was revealed for the trend test when analyzed by study. For smoking duration, we found a reduction in risk increase for smoking 11–20 years from 20% (HR = 1.20, 95% CI 1.03–1.40) to nonsignificant 6% (HR = 1.06, 95% CI 0.89–1.27) for smoking ≥21 years. A subanalysis for high number of pack-years as a measure of high lifetime smoking exposure showed a nonsignificant increase in breast cancer mortality of only 3% (HR = 1.03, 95% CI 0.79–1.34) for ≥21 pack-years (66 cases).

**Table 3:** Mulitvariate adjusted hazards ratios (HRs) and 95% confidence intervals (CIs) for breast cancer mortality^1^, stratified by studies, with never smokers as the reference group

Smoking exposures	Person Years (ALL)	Cases (ALL)	Counties Study	40 Years Study	CONOR Study	All studies
Smoking status						
Never	1,733,948	459	**Ref.**			
Ever	2,430,366	647	1.18, (0.97–1.44)	1.15 (0.97–1.36)	1.26 (0.78–2.03)	1.15–(1.02–1.30)
Former	819,115	216	1.06 (0.79–1.44)	1.17 (0.95–1.45)	1.39 (0.81–2.38)	1.14 (0.97–1.34)
Current	1,611,251	431	1.23 (0.99–1.52)	1.13 (0.94–1.36)	1.11 (0.60–2.04)	1.15 (1.01–1.32)
		Cases	405	624	77	1106
Ever smokers						
Age at smoking initiation (years)						
≥25	355,209	137	1.33 (1.00–1.77)	1.26 (0.95–1.67)	1.28 (0.61–2.69)	1.31 (1.08–1.59)
20–24	585,167	149	1.03 (0.74–1.43)	1.10 (0.86–1.40)	0.85 (0.37–1.93)	1.04 (0.86–1.26)
≤19	921,599	182	1.36 (0.98–1.88)	1.00 (0.78–1.27)	1.37 (0.75–2.50)	1.05 (0.87–1.25)
	Sum	468	*P*_trend_ 0.082	*P*_trend_ 0.11	*P*_trend_ 0.66	*P*_trend_ 0.052
Smoking duration^2^ (years)						
≤10	702,018	178	1.24 (0.94–1.63)	1.08 (0.85–1.38)	1.40 (0.68–2.91)	1.13 (0.95–1.35)
11–20	1,008,418	291	1.17 (0.91–1.49)	1.23 (1.01–1.51)	0.91 (0.41–2.02)	1.20 (1.03–1.40)
≥21	703,524	178	1.12 (0.79–1.59)	1.05 (0.83–1.32)	1.27 (0.71–2.26)	1.06 (0.89–1.27)
	Sum	647	*P*_trend_ 0.23	*P*_trend_ 0.27	*P*_trend_ 0.53	*P*_trend_ 0.43
Number of cigarettes per day						
≤5	438,860	126	1.00 (0.71–1.39)	1.37 (1.06–1.78)	1.17 (0.57–2.38)	1.18 (0.97–1.43)
6–10	1,071,909	263	1.04 (0.81–1.34)	1.08 (0.88–1.33)	1.17 (0.63–2.16)	1.06 (0.91–1.23)
11–15	553,968	161	1.65 (1.22–2.24)	1.13 (0.89–1.45)	0.70 (0.24–2.00)	1.25 (1.04–1.51)
≥16	350,559	97	1.53 (1.00–2.33)	1.08 (0.81–1.43)	2.53 (1.18–5.43)	1.24 (0.99–1.55)
	Sum	647	*P*_trend_ <0.01	*P*_trend_ 0.50	*P*_trend_ 0.27	*P*_trend_ 0.26
Number of pack-years^3^						
≤5	811,590	211	1.09 (0.84–1.41)	1.15 (0.92–1.44)	1.52 (0.82–2.85)	1.12 (0.95–1.32)
6–10	650,196	183	1.12 (0.84–1.48)	1.18 (0.93–1.50)	0.84 (0.35–2.04)	1.14 (0.95–1.36)
11–15	474,227	132	1.45 (1.04–2.01)	1.13 (0.80–1.46)	1.08 (0.44–2.63)	1.22 (1.00–1.49)
≥16	468,639	121	1.35 (0.89–2.06)	1.07 (0.83–1.39)	1.30 (0.65–2.62)	1.13 (0.92–1.39)
	Sum	647	*P*_trend_ 0.13	*P*_trend_ 0.94	*P*_trend_ 0.78	*P*_trend_ 0.61
Smoking duration before first childbirth among parous women (years)						
After first childbirth (>1 year)	362,485	104	1.15 (0.83–1.58)	0.94 (0.69–1.29)	0.84 (0.29–2.38)	1.03 (0.83–1.28)
Around childbirth^4^	274265	62	1.11 (0.71–1.72)	0.84 (0.59–1.21)	1.58 (0.55–4.56)	0.94 (0.72–1.24)
>1–6 years before	629,381	157	1.32 (0.95–1.84)	1.22 (0.95–1.56)	1.00 (0.49–2.03)	1.17 (0.97–1.41)
≥7 years before	388,480	89	1.56 (0.99–2.76)	1.24 (0.91–1.70)	1.27 (0.64–2.55)	1.24 (0.98–1.58)
	Sum	412	*P*_trend_^5^ 0.73	*P*_trend_^5^ 0.60	*P*_trend_^5^ 0.68	*P*_trend_^5^ 0.47

Adjusted for age, education level, number of children, age at first childbirth, BMI, age during enrollment and physical activity.

Trend tests between the levels of smoking categories excluding never smokers, except otherwice noted.

1 For breast cancer deaths (*n* = 302,865 with 1106 cases).

2 Total number of years smoked.

3 Pack years: Number of cigarettes smoked per day multiplied by number of years smoked. One pack has 20 cigarettes.

4 1 year before to 1 year after first childbirth.

5 Trend tests between three levels of smoking categories (around, >1–6, ≥7), excluding never smokers and smoking after first childbirth.

We also did a stratified analysis by birth cohorts (≤1950>), including 149,270 and 878 breast cancer deaths for those born in and before 1950, and 153,595 women and 228 breast cancer deaths in those born after 1950. We found a 5% difference in breast cancer mortality risk for ever smokers as compared with never smokers, for the oldest birth cohort (HR = 1.15, 95% CI 1.00–1.32) and the corresponding figure for those born after 1950 (HR = 1.20, 95% CI 0.90–1.59). The test for heterogeneity revealed no significant difference (*P*_Wald_ = 0.80). Comparing the highest number of pack-years (≥11 pack-years) between these birth cohorts showed a 3% risk difference (HR = 1.20, 95% CI 1.00–1.43) and (HR = 1.17, 95% CI 0.82–1.66), respectively.

Stratification by birth cohort (≤1950>) showed a 15% difference in breast cancer mortality for ever smokers as compared with never smokers, for those born in 1950 or before (HR = 1.15, 95% CI 1.00–1.32) and the corresponding figure for those born after 1950 (HR = 1.30, 95% CI 0.93–1.80). The test for heterogeneity revealed no significant difference (*P*_Wald_ = 0.52). Stratification for the other exposure variables (age at smoking initiation, smoking duration, number of cigarettes smoked per day, number of pack-years and smoking duration before first childbirth) by birth cohort (≤1950>) all showed nonsignificant results (data not shown).

We included alcohol consumption as a covariate in the multivariate model after excluding women without alcohol information, restricting the sample size to 114,804 women. All the results became statistically nonsignificant. For ever smokers as compared with never smokers, the estimate for breast cancer mortality increased to 20% (HR = 1.20, 95% CI 0.82–1.75). For former smokers the corresponding figure increased to 26% (HR = 1.26, 95% CI 0.82–1.96), and for current smokers it decreased to 13% (HR = 1.13, 95% CI 0.73–1.76). A Wald's test for heterogeneity between the subpopulation with alcohol information and the full analytical cohort yielded nonsignificant results for ever (*P*_Wald_ = 0.84), current (*P*_Wald_ = 0.93) and former smokers (*P*_Wald_ = 0.67).

## Discussion

Overall, we observed a slight increase in breast cancer mortality among ever smokers compared to never smokers, with similar results when the analyses were stratified by the three studies, and by birth cohort. No statistically significantly increased mortality was found for women who initiated smoking before first childbirth, and no dose-response association was revealed for any of the different measures of smoking exposure (age at smoking initiation, smoking duration, number of cigarettes smoked per day, number of pack-years, or smoking duration before first childbirth).

Our finding of an increased smoking-related risk of breast cancer mortality, but without a dose-response association between the exposure and outcome variables, is probably a result of competing risks of mortality, as heavy and long-term smokers could have died from other smoking-related diseases 45,46. Also, the excellent survival of breast cancer patients in Norway could make this association more difficult to study 3.

The smoking pattern in Norway 47, as in other countries 48, has changed considerably in recent generations, with a high smoking prevalence in the birth cohorts during and just after World War II. Our previous study showed an increased breast cancer incidence among ever smokers as compared to never smokers, particularly among those initiating smoking before the first childbirth, as for other exposures 19. These increases in risk were not replicated for breast cancer mortality in the present analysis due to lack of significant dose-response results.

Most previous studies examining the association between smoking and death from breast cancer have assessed smoking status at breast cancer diagnosis without consideration of lifetime smoking exposure 25–32, disregarding the fact that the hazards of smoking are often accumulated with exposure 6, and that the period before, and not after, breast cancer diagnosis is essential in respect to carcinogenesis.

The report by Pirie et al. from the Million Women Study found a 13% significantly increased risk for breast cancer mortality associated with current smoking 22. The study also performed sensitivity analyses for breast cancer mortality among nondrinking women, reducing the increase in risk from a significant 13% to a nonsignificant 6%. Our sensitivity analyses, which included only women with information on alcohol consumption (*n* = 114,804), increased the risk for ever and former smokers to 20% and 26%, respectively, and reduced the risk for current smokers to 13%, as compared to the main analysis. The tests for heterogeneity for smoking status (ever, former, current), between the populations with and without alcohol information revealed no significant difference between the results for breast cancer mortality, suggesting that alcohol consumption is of limited importance in this study. However, higher alcohol consumption is a known risk factor for overall mortality 49, and may have diluted our results by increasing competing risks during follow-up. The paper from the US Cancer Prevention Study II (1994) found a 26% statistically significantly increased breast cancer mortality among current smokers, and a nonsignificantly reduced mortality among former smokers, as compared to never smokers 21. In 2013, the short report from the US Women's Healthy Eating and Living (WHEL) study including 2953 women and 245 breast cancer deaths during 7.3 years of follow-up found a nonsignificantly increased breast cancer mortality among current and former smokers reported at breast cancer diagnosis. In the same study, the analysis was performed by high-smoking exposure *before* breast cancer diagnosis as a proxy for lifetime smoking exposure, finding a significantly increased mortality risk of 54% for >20 pack-years 23. Another study included three US cohorts with 1059 breast cancer deaths and a mean exposure of 39 pack-years for current smokers. This study found 54% significantly increased breast cancer mortality among former smokers with a very high lifetime smoking exposure of more than 35 pack-years. No increase was found in those with less smoking exposure 24. Our analysis for high smoking exposure showed that the increased breast cancer mortality was reduced from 17% for those smoking ≥11 pack-years to 3% for those smoking ≥21 pack-years. Similarly, the risk was reduced from 20% for smoking 11–20 years to 6% for smoking ≥21 years. A reduction in breast cancer mortality for women with the highest smoking exposure may be explained by a greater impact from competing risks in heavy smokers as compared with less heavy smokers in this cohort, but contrasts with the recent study from the US showing increased breast cancer mortality with very high exposure 24. Further, current smokers in this study had a mean exposure of 13 pack-years, which is lower than the US study, and may explain some of the conflicting results for the highest smoking exposure categories. Taken together, the previously conducted studies may suggest a causal relationship between high- lifetime smoking exposure and breast cancer mortality, but this study could not confirm these results.

To our knowledge, no previous studies have examined in detail the association with smoking before first childbirth and breast cancer mortality, as presented in this paper. We consider it a major strength that smoking assessment in this study was conducted with smoking status at enrolment, and not at breast cancer diagnosis, to show the true risk between lifetime smoking exposure and breast cancer death. Other strengths include its prospective, nationwide population-based cohort design, the large size and the complete follow-up through national registries. Our analytical cohort included a large proportion of young smoking females, many of whom initiated smoking before first childbirth, which enabled us to study this period in detail. Smoking histories were obtained at enrolment and were not subject to recall bias. Other studies have found substantial differences in smoking status in studies with long follow-up 22,27. Our data includes smoking information at study enrolment only, and our multivariate analysis by smoking status is between ever and never smokers, leaving never smokers as the only category that could possibly change status in the follow-up period. This reduces the chance of misclassification bias in the follow-up period. The smoking pattern in the Norwegian population was stable in the follow-up period 1974–2007 with about 32% female current smokers 47. Also, since very few Norwegians start to smoke after the age of 30 and the mean age at enrolment in this study is 44 years, we do not expect changes in smoking status among the never smokers to influence our risk estimates.

Our study also has important limitations: we were not able to adjust for some covariates which may increase breast cancer incidence, such as the use of hormone replacement therapy, use of oral contraceptives, family history of breast cancer, higher mammographic density, higher age at menopause, lower age at menarche, weight gain during adult life, or alcohol consumption 50,51. We used duration of education as a proxy for socioeconomic status 52. The Norwegian Breast Cancer Screening Programme and contemporary breast clinics were established nationwide only 2 years before the end of the follow-up of this study, and therefore we find it unlikely that this has biased our results. Information about age at smoking initiation was not reported in two of the three studies and was calculated from other information reported in the questionnaires, which may have introduced bias in any of the estimations of timing relative to first childbirth. Using ever smokers in the analysis instead of current and former smokers makes it impossible to distinguish between current smoking, often used as a surrogate for heavy smoking exposure, and former smoking, often with a disparate smoking exposure history. Furthermore, around 10% of the female Norwegian population has reported being occasional smokers during the last four decades 47, but occasional and passive smokers were probably included in never smokers in our study, since we did not ask specific questions about these factors. Stage at diagnosis was not included as a confounder in this study, but since our analysis is based on information before diagnosis we do not consider this as a major limitation. We lack complete data on other causes of death, and we made no further attempts to assess other competing risks of mortality. Finally, higher comorbidity is a known adverse prognostic factor for death after breast cancer diagnosis 53, and a large number of such competing risks may have changed the associations in this study towards the null, as women have died from other causes than breast cancer during follow-up 45,46.

Although still controversial 9, the rationale for an increased breast cancer risk in women initiating smoking before first childbirth is reported in many biological 37,54 and epidemiological studies 7,8,14,16–19,36, but the increased risk of dying from breast cancer has not been previously assessed for this category. This study found that lifetime smoking exposure had a significantly increased risk of breast cancer mortality compared with never smokers, but without clear dose-response associations. Further studies are needed, possibly in cohorts with very high smoking exposure, in order to conclude an association between lifetime smoking exposure and breast cancer mortality.

## References

[1] Kalager M, Zelen M, Langmark F, Adami HO (2010). Effect of screening mammography on breast-cancer mortality in Norway. N. Engl. J. Med.

[2] Olsen AH, Lynge E, Njor SH, Kumle M, Waaseth M, Braaten T (2013). Breast cancer mortality in Norway after the introduction of mammography screening. Int. J. Cancer.

[3] Cancer Registry of Norway (2013).

[4] Marmot MG, Altman DG, Cameron DA, Dewar JA, Thompson SG, Wilcox M (2013). The benefits and harms of breast cancer screening: an independent review. Br. J. Cancer.

[5] Ferlay J, Steliarova-Foucher E, Lortet-Tieulent J, Rosso S, Coebergh JW, Comber H (2013). Cancer incidence and mortality patterns in Europe: estimates for 40 countries in 2012. Eur. J. Cancer.

[6] IARC (2012). International agency for research on cancer IARC monograph 100E: personal habits and indoor combustions. A review of human carcinogens.

[7] Johnson KC, Miller AB, Collishaw NE, Palmer JR, Hammond SK, Salmon AG (2011). Active smoking and secondhand smoke increase breast cancer risk: the report of the Canadian expert panel on tobacco smoke and breast cancer risk (2009). Tob. Control.

[8] DeRoo LA, Cummings P, Mueller BA (2011). Smoking before the first pregnancy and the risk of breast cancer: a meta-analysis. Am. J. Epidemiol.

[9] U.S. Department of Health and Human Services (2014).

[10] Glantz SA, Johnson KC (2014). The surgeon general report on smoking and health 50 years later: breast cancer and the cost of increasing caution. Cancer Epidemiol. Biomarkers Prev.

[11] Reynolds P, Hurley S, Goldberg DE, Anton-Culver H, Bernstein L, Deapen D (2004). Active smoking, household passive smoking, and breast cancer: evidence from the California Teachers Study. J. Natl. Cancer Inst.

[12] Al-Delaimy WK, Cho E, Chen WY, Colditz G, Willet WC (2004). A prospective study of smoking and risk of breast cancer in young adult women. Cancer Epidemiol. Biomarkers Prev.

[13] Olson JE, Vachon CM, Vierkant RA, Sweeney C, Limburg PJ, Cerhan JR (2005). Prepregnancy exposure to cigarette smoking and subsequent risk of postmenopausal breast cancer. Mayo Clin. Proc.

[14] Gram IT, Braaten T, Terry PD, Sasco AJ, Adami HO, Lund E (2005). Breast cancer risk among women who start smoking as teenagers. Cancer Epidemiol. Biomarkers Prev.

[15] Cui Y, Miller AB, Rohan TE (2006). Cigarette smoking and breast cancer risk: update of a prospective cohort study. Breast Cancer Res. Treat.

[16] Ha M, Mabuchi K, Sigurdson AJ, Freedman DM, Linet MS, Doody MM (2007). Smoking cigarettes before first childbirth and risk of breast cancer. Am. J. Epidemiol.

[17] Xue F, Willett WC, Rosner BA, Hankinson SE, Michels KB (2011). Cigarette smoking and the incidence of breast cancer. Arch. Intern. Med.

[18] Gaudet MM, Gapstur SM, Sun J, Diver WR, Hannan LM, Thun MJ (2013). Active smoking and breast cancer risk: original cohort data and meta-analysis. J. Natl. Cancer Inst.

[19] Bjerkaas E, Parajuli R, Weiderpass E, Engeland A, Maskarinec G, Selmer R (2013). Smoking duration before first childbirth: an emerging risk factor for breast cancer? Results from 302,865 Norwegian women. Cancer Causes Control.

[20] Rosenberg L, Boggs DA, Bethea TN, Wise LA, Adams-Campbell LL, Palmer JR (2013). A prospective study of smoking and breast cancer risk among African-American women. Cancer Causes Control.

[21] Calle EE, Miracle-McMahill HL, Thun MJ, Heath CW (1994). Cigarette smoking and risk of fatal breast cancer. Am. J. Epidemiol.

[22] Pirie K, Peto R, Reeves GK, Green J, Beral V (2013). The 21st century hazards of smoking and benefits of stopping: a prospective study of one million women in the UK. Lancet.

[23] Saquib N, Stefanick ML, Natarajan L, Pierce JP (2013). Mortality risk in former smokers with breast cancer: pack-years vs. smoking status. Int. J. Cancer.

[24] Pierce JP, Patterson RE, Senger CM, Flatt SW, Caan BJ, Natarajan L (2014). Lifetime cigarette smoking and breast cancer prognosis in the after breast cancer pooling project. J. Natl. Cancer Inst.

[25] Manjer J, Andersson I, Berglund G, Bondesson L, Garne JP, Janzon L (2000). Survival of women with breast cancer in relation to smoking. Eur. J. Surg.

[26] Fentiman IS, Allen DS, Hamed H (2005). Smoking and prognosis in women with breast cancer. Int. J. Clin. Pract.

[27] Holmes MD, Murin S, Chen WY, Kroenke CH, Spiegelman D, Colditz GA (2007). Smoking and survival after breast cancer diagnosis. Int. J. Cancer.

[28] Sagiv SK, Gaudet MM, Eng SM, Abrahamson PE, Shantakumar S, Teitelbaum SL (2007). Active and passive cigarette smoke and breast cancer survival. Ann. Epidemiol.

[29] Barnett GC, Shah M, Redman K, Easton DF, Ponder BA, Pharoah PD (2008). Risk factors for the incidence of breast cancer: do they affect survival from the disease?. J. Clin. Oncol.

[30] Hellmann SS, Thygesen LC, Tolstrup JS, Gronbaek M (2010). Modifiable risk factors and survival in women diagnosed with primary breast cancer: results from a prospective cohort study. Eur. J. Cancer Prev.

[31] Braithwaite D, Izano M, Moore DH, Kwan ML, Tammemagi MC, Hiatt RA (2012). Smoking and survival after breast cancer diagnosis: a prospective observational study and systematic review. Breast Cancer Res. Treat.

[32] Warren GW, Kasza KA, Reid ME, Cummings KM, Marshall JR (2013). Smoking at diagnosis and survival in cancer patients. Int. J. Cancer.

[33] Norges offentlige utredninger (2000). Tobakksindustriens erstatningsansvar. [Norway's public reports. Tobacco Industry Liability].

[34] Elstad JI, Torstensrud R, Lyngstad TH, Kravdal O (2012). Trends in educational inequalities in mortality, seven types of cancers, Norway 1971–2002. Eur. J. Public Health.

[35] Russo J, Moral R, Balogh GA, Mailo D, Russo IH (2005). The protective role of pregnancy in breast cancer. Breast Cancer Res.

[36] Lawlor DA, Ebrahim S, Smith GD (2004). Smoking before the birth of a first child is not associated with increased risk of breast cancer: findings from the British Women's Heart and Health Cohort Study and a meta-analysis. Br. J. Cancer.

[37] Russo J, Russo IH (1999). Cellular basis of breast cancer susceptibility. Oncol. Res.

[38] Stocks T, Borena W, Strohmaier S, Bjorge T, Manjer J, Engeland A (2010). Cohort profile: the metabolic syndrome and cancer project (me-can). Int. J. Epidemiol.

[39] Bjartveit K, Foss OP, Gjervig T, Lund-Larsen PG (1979). The cardiovascular disease study in Norwegian counties. Background and organization. Acta Med. Scand. Suppl.

[40] Bjartveit K, Stensvold I, Lund-Larsen PG, Gjervig T, Kruger O, Urdal P (1991). Cardiovascular screenings in Norwegian counties. Background and implementation. Status of risk pattern during the period 1986–90 among persons aged 40–42 years in 14 counties. Tidsskr. Nor. Laegeforen.

[41] Naess O, Sogaard AJ, Arnesen E, Beckstrom AC, Bjertness E, Engeland A (2008). Cohort profile: Cohort of Norway (CONOR). Int. J. Epidemiol.

[42] Larsen IK, Smastuen M, Johannesen TB, Langmark F, Parkin DM, Bray F (2009). Data quality at the cancer registry of Norway: an overview of comparability, completeness, validity and timeliness. Eur. J. Cancer.

[43] Alfsen GC, Maehlen J (2012). The value of autopsies for determining the cause of death. Tidsskr. Nor. Laegeforen.

[44] Hofvind S, Ursin G, Tretli S, Sebuodegard S, Moller B (2013). Breast cancer mortality in participants of the Norwegian Breast Cancer Screening Program. Cancer.

[45] Thun M, Peto R, Boreham J, Lopez AD (2012). Stages of the cigarette epidemic on entering its second century. Tob. Control.

[46] Gram IT, Sandin S, Braaten T, Lund E, Weiderpass E (2013). The hazards of death by smoking in middle-aged women. Eur. J. Epidemiol.

[47] Lund M, Lindbak R (2007).

[48] Pierce JP, Messer K, White MM, Cowling DW, Thomas DP (2011). Prevalence of heavy smoking in California and the United States, 1965–2007. JAMA.

[49] Baan R, Straif K, Grosse Y, Secretan B, El GF, Bouvard V (2007). Carcinogenicity of alcoholic beverages. Lancet Oncol.

[50] Key TJ, Verkasalo PK, Banks E (2001). Epidemiology of breast cancer. Lancet Oncol.

[51] Li CI (2010). Breast Cancer Epidemiology.

[52] Huisman M, Kunst AE, Bopp M, Borgan JK, Borrell C, Costa G (2005). Educational inequalities in cause-specific mortality in middle-aged and older men and women in eight western European populations. Lancet.

[53] Land LH, Dalton SO, Jensen MB, Ewertz M (2012). Influence of comorbidity on the effect of adjuvant treatment and age in patients with early-stage breast cancer. Br. J. Cancer.

[54] Russo J, Russo IH (1994). Toward a physiological approach to breast cancer prevention. Cancer Epidemiol. Biomarkers Prev.

